# Is laboratory screening prior to antiretroviral treatment useful in Johannesburg, South Africa? Baseline findings of a clinical trial

**DOI:** 10.1186/s12889-017-4353-1

**Published:** 2017-07-04

**Authors:** Willem D. F. Venter, Mohammed Majam, Godspower Akpomiemie, Natasha Arulappan, Michelle Moorhouse, Nonkululeko Mashabane, Matthew F. Chersich

**Affiliations:** Wits Reproductive Health and HIV Institute (WRHI), Faculty of Health Sciences, University of the Witwatersrand, Johannesburg, South Africa

**Keywords:** South Africa, HIV, Antiretroviral treatment, Screening, Laboratory, Hepatitis B

## Abstract

**Background:**

Screening for renal, hepatic and haematological disorders complicates the initiation of current first-line antiretroviral therapy (ART). Each additional test done adds substantial costs, both through direct laboratory expenses, but also by increasing the burden on health workers and patients. Evaluating the prevalence of clinically relevant abnormalities in different population groups could guide decisions about what tests to recommend in national guidelines, or in local adaptations of these.

**Methods:**

As part of enrolment procedures in a clinical trial, 771 HIV-positive adults, predominantly from inner-city primary health care clinics, underwent laboratory screening prior to ART. Participants had to be eligible for ART, based on the then CD4 eligibility threshold of 350 cells/μL, antiretroviral naïve and have no symptoms of peripheral neuropathy.

**Results:**

Participants were mostly female (57%) and a mean 34 years old. Creatinine clearance rates were almost all above 50 mL/min (99%), although 5% had microalbuminuria. Hepatitis B antigenaemia was common (8% of participants), of whom 40% had a raised AST/ALT, though only 2 had transaminase levels above 200 IU/L. Only 2% of participants had severe anaemia (haemoglobin <8 g/dl) and 1% neutropaenia (neutrophils <0.75 × 10^9/L). Costs per case detected of hepatitis B infection was USD135, but more than USD800 for a raised creatinine.

**Conclusions:**

Hepatitis B continues to be a common co-infection in HIV-infected adults, and adds complexity to management of ART switches involving tenofovir. Routine renal and haematological screening prior to ART detected few abnormalities. The use of these screening tests should be assessed among patients with higher CD4 counts, who may even have fewer abnormalities. Formal evaluation of cost-effectiveness of laboratory screening prior to ART is warranted.

## Background

The trend over the past decade has been to initiate antiretroviral therapy (ART) in HIV-positive patients at higher CD4 counts: ART initiation thresholds in WHO guidelines were 350 cells/μL in 2010, 500 cells/μL in 2013, and then ultimately treatment was recommended for all HIV-infected patients in 2015 [1, 2]. With these changes and increased coverage of HIV testing, numbers of people receiving ART have increased exponentially. These increases mean that these services must be simplified wherever possible. Tenofovir (TDF) and emtricitabine (FTC) or lamivudine (3TC), in combination with efavirenz (EFV), is recommended by WHO as first-line ART in countries that can afford it, with recommendations on screening for renal disease prior to TDF use [2]. This regimen (TDF/FTC/EFV) is currently used as first-line ART in state programmes in South Africa (a single tablet, fixed-dose formulation taken once daily).

The major side effect of TDF is renal failure, secondary to tubular dysfunction [3]. This is uncommon and usually reversible, but can be catastrophic for individual patients who experience severe renal failure, as renal replacement therapy (dialysis, transplant) is seldom available in poorer regions of the country [4]. Existing renal dysfunction is a risk factor for TDF toxicity [5]. Toxicity may also be compounded by the concomitant usage of other nephrotoxic drugs taken as treatment for common infective complications, such as cryptococcal meningitis and multi-drug resistant tuberculosis [4, 5]. WHO and South Africa Department of Health recommendations to decrease TDF toxicity include an assessment of creatinine clearance prior to initiation with ART, with subsequent follow-up monitoring of clearance and the use of alternative antiretrovirals if abnormalities progress over time [2, 6].

TDF in combination with FTC, or its analogue 3TC, is also a potent suppressor of hepatitis B, which is common in the general adult Southern African population [2, 7]. Routine hepatitis B vaccination was only introduced into South Africa in 1995, meaning that still large numbers of adults have the infection [8]. Even though TDF-containing regimens are recommended in these patients, hepatitis B screening is still recommended in many guidelines [6, 9], as it may firstly predict for immune reconstitution syndromes (liver ‘flares’ may occur during the first weeks and months of treatment with any ART regimen), and secondly, as an interruption of suppressive TDF/3TC (or FTC) for whatever reason may cause a viral hepatitis, which occasionally can be fatal [2, 10–12]. In addition to screening for renal dysfunction, laboratory screening prior to ART initiation often includes a full blood count. Anaemia and neutropaenia are frequent findings in patients with advanced HIV, and both are relative contra-indications for zidovudine (AZT), which is commonly used in many places if TDF is contraindicated.

A group of patients presenting for routine HIV care in central Johannesburg, had biomedical tests done as part of screening for a clinical trial. These tests documented the prevalence of renal disease, hepatitis B (and correlation with abnormal alanine aminotransferase [ALT] or aspartate aminotransferase [AST]), anaemia and neutropaenia. Information on the frequency of abnormal results on these tests, which are used for screening prior to ART, could help inform decisions about the usefulness of these tests are in our population and similar groups elsewhere.

## Methods

Antiretroviral naïve adults attending outpatient HIV testing and treatment services in central Johannesburg were invited to participate in a clinical trial (WRHI 001) [13] by community workers. At the time of the study, the CD4 initiation threshold in South Africa was 350 cells/μL. Potential participants were approached in inner-city clinics in Hillbrow or, less commonly, within a tertiary-level facility, the Charlotte Maxeke Johannesburg Academic Hospital (CMJAH), where the study was based. Community workers used a checklist to identify potential participants, based on the study inclusion criteria, which included age above 18 years, no previous exposure to antiretroviral drugs from preventing mother-to-child transmission or as ART, CD4 counts <400 cells/μL, and women who were not pregnant or breastfeeding. The trial explored two alternative first-line antiretroviral regimens: tenofovir versus low-dose stavudine (20 mg twice daily), in patients also receiving lamivudine and efavirenz. The study had sites in Uganda and India, but data presented here are only for the South African site.

### Study procedures

Once informed consent processes were completed, blood and a urine sample were obtained. Laboratory procedures included a full blood count, CD4 cell count, creatinine clearance, microalbuminuria measurement, urine dipstick, hepatitis B antigen test, liver function test and an HIV plasma viral load. CD4 testing was done using Beckman Coulter FC500 machines.

### Study indicators and data analysis

Participants’ demographics and health status (WHO clinical stage, CD4 count and viral load results) were used to describe the study population. The remainder of the indicators assess the prevalence of renal, hepatic and haematological disease in the population. A creatinine clearance <50 mL/min was categorised as abnormal, as it is the recommended cut-off for use of TDF according to national guidelines [6]. Microalbuminuria was defined as a microalbumin-to-creatinine ratio of 3.4–33.9 mg/mmol, while a ratio above 34.0 mg/mmol was classified as proteinuria. The prevalence of hepatitis B antigenaemia and of abnormal ALT and AST are reported. Levels of AST and ALT above 40 U/L were defined as abnormal, and reported together with the proportion above 100 U/L (2.5 times normal) or above 200 U/L (5 times normal), as relevant. A haemoglobin <10.0 g/dL was considered anaemia and <8.0 g/dL severe anaemia. Participants with neutrophil levels <0.75 × 10^9/L were defined as being neutropenic. Platelet counts (cells/μl) were categorised as <50,000, 50,000–125,000 and above 125,000, drawing on categories in the Division of AIDS grading of adverse events [14]. We also estimated the US dollar (USD) laboratory costs per detection of a case of hepatitis B virus (USD10.7/test), or of abnormal creatinine clearance (USD4.3/test), microalbumin (USD6.1/test), AST or ALT (USD4.3/test) in our population (Rand13.3 = 1USD), but did not conduct a formal cost-effectiveness assessment.

## Results

### Participant demographics and health status

Most of the 771 patients screened for trial eligibility were from inner-city clinics in Hillbrow Johannesburg (93%), while 7% were from the nearby CMJAH hospital. Population characteristics, health status and results of laboratory screening tests are summarised in Table 1. Participants were all Black Africans, mostly female (57%) and a mean 33.9 years old (standard deviation = 7.7). Twelve percent were older than 45 years (*n* = 91). More than 40% of the cohort were from outside South Africa, predominantly from Zimbabwe (38% of participants). Three quarters were employed and the same proportion described themselves as being single. Around a third reported current alcohol use (36%) and 16% smoke. Only 3% of the participants had WHO Stage IV disease and 16% Stage III conditions. CD4 cell counts at screening were a median 208 cells/μL (inter-quartile range = 118–299), with only 8% having a count below 50 cells/μL and 13% above 350. Plasma viral load varied markedly between patients, with 18% having fewer than 10,000 copies per ml, while 39% had counts above 100,000.

**Table 1:** Demographics and laboratory data of screened patients in the WRHI001 South African Cohort

Variable	Proportion or central measure
*Population demographics and health status*	
Female n (%)	432 (57%)
Mean age (sd)	33.9 (7.7)
Nationality n (%)	
South African	431 (57%)
Zimbabwean	287 (38%)
Other African countries	40 (5%)
Marital status n (%)	
Married	174 (24%)
Divorced or separate	12 (2%)
Single	554 (75%)
Employed n (%)	551 (75%)
Current alcohol use n (%)	269 (36%)
Current smoking n (%)	116 (16%)
WHO clinical stage	
I	433 (59%)
II	164 (22%)
III	119 (16%)
IV	20 (3%)
Median CD4 cells/μL at screening (IQR), *n* = 755^a^	208 (118-299)
Median plasma viral load copies/mL (IQR)	64,782 (17,646–186,662)
*Laboratory screening tests prior to antiretroviral treatment*	
Creatinine clearance *n* < 50 mL/min (%)	4 (1%)
Microalbumin-to-creatinine ratio mg/mmol n (%)	
3.4–33.9	40 (5%)
> 34.0	2 (0%)
Urine dipstick n (%)	
Proteinuria 1+	60 (8%)
Proteinuria 2+	14 (2%)
Proteinuria 3+	7 (1%)
Hepatitis B surface antigen positive n (%)	60 (8%)
Abnormal ALT/AST IU/L n (%) in all patients	
40–99 (1-2.4 X normal)	145 (19%)
100–199 (2.5-4.9 X normal)	22 (3%)
≥ 200 (≥5 X normal)	4 (1%)
Abnormal ALT/AST IU/L n (%) If hepatitis B positive	
40–99	19 (32%)
100–199	2 (3%)
≥ 200	2 (3%)
Anaemia in females n (%)	
8–10.0 g/dL	40 (9%)
< 8.0 g/dL	10 (2%)
Anaemia in males n (%)	
8–10.0 g/dL	13 (4%)
< 8.0 g/dL	2 (1%)
Neutrophil count	
< 0.75 × 10^9 /L n (%)	7 (1%)
Median cells (IQR)	2.2 (1.6-2.9)
Platelet counts (cells/μl) n (%)	
< 50,000	3 (0)
50,000--125,000	25 (3)

^a^16 patients were excluded from the trial for reasons other than CD4 count, and thus CD4 tests were not done. IQR - inter-quartile range. sd - standard deviation

### Laboratory screening tests

One percent of patients had a creatinine clearance below 50 mL/min. The CD4 cells/μL for these four patients were 128, 306, 293 and 131. Microalbuminuria was not infrequent, occurring in 5% of patients. Prevalence of proteinuria was, however, very low, measured either by microalbuminuria or 3+ proteins on urine dipstick. Prevalence of hepatitis B antigenaemia was 8%. Almost 40% of patients with hepatitis B had a raised transaminase, of whom two patients had an ALT or AST above 200 IU/L (one had an AST of 842 and ALT of 686, while the AST was 285 and ALT 611 in the other). Among the whole cohort, mild elevations of ALT/AST levels (1-2.4 fold the upper limit of normal) were relatively frequent (19%), and a further 4% had even higher levels.

The mean haemoglobin was 12.0 g/dL (sd = 1.7) in women and 13.9 g/dL (sd = 2.0) in men (*p* ≤ 0.001). Overall, 9% of patients had anaemia and 2% severe anaemia, with the frequency of anaemia in women double that of men. Only 1% of participants had neutropaenia (neutrophils <0.75 × 10^9/L). Four percent of participants had a platelet count below 125,000 cells/μl, and these were mostly around the 100,000 mark. The costs in our population of identifying one case of hepatitis B virus was USD135, and one abnormal result for creatinine clearance USD820, microalbumin USD18, AST or ALT above 40 USD19, and AST or ALT above 100 USD126.

Finally, a total of 600 patients met study inclusion criteria and were enrolled. Patients were largely excluded on the basis of laboratory tests, mostly due to: hepatitis B positive (60), CD4 count >350 cells/μL (96), elevated creatinine clearance (5), anaemia (4), thrombocytopaenia (5), and elevated AST/ALT level (3). Some patients had multiple reasons for study exclusion.

## Discussion

The study showed that relatively few patients had abnormal results on the tests used for screening for ART eligibility in South Africa. The prevalence of abnormal tests may be even lower among the relatively more healthy cohorts who are initiating ART presently, given that all HIV-infected patients are now eligible for ART, regardless of CD4 count. In particular, our findings raise questions about the utility of routine creatinine clearance testing before initiating TDF in our setting. Significant renal dysfunction was very uncommon in our study, as measured by creatinine clearance, although microalbuminuria was relatively frequent. As further data accrues on this topic, ART guideline committees could consider minimising the number of tests done to identify relative and absolute contraindications to the use of certain antiretrovirals. Alternatively, future guideline iterations may consider continuing the screening tests, but recommending that ART could be initiated while waiting for the results and then patients regimens be altered at the subsequent visit, if required.

Findings from several other South African cohorts on creatinine clearance have been conflicting, with most also reporting low levels of renal dysfunction, but a few recording abnormal results in as many as 5% of patients initiating antiretrovirals [15–17]. All these findings, however, are far lower than other reports from the rest of the continent [3, 18]. The urine abnormalities at ART initiation may reflect concurrent illness, rather than renal dysfunction per se; in addition, some renal dysfunction may not be measured by creatinine clearance. Moreover, a retrospective record review in 2014 in Lusaka, looking at patients who initiated TDF while having renal dysfunction, suggested that most patients with moderate to severe abnormal creatinine clearances do not experience deterioration of renal function on TDF [19]. Similarly, previous data from a study in our site suggest that most urine abnormalities tend to normalise over time [20]. Based on the above factors, we contend that renal screening with urine dipstick or creatinine clearance may add little to patient management in our setting, and unnecessarily delay ART initiation, while increasing programme complexity and cost. Prospective analysis of the treated cohort described here, where proteinuria and microalbuminuria will be followed over time, may help confirm that assertion.

Hepatitis B infection was common, in keeping with previous reports, both from the general population, as well as from other HIV clinics in South Africa [21–23]. It is unclear how commonly hepatic flares or withdrawal hepatitis occurs with ART [23], as currently data are limited to isolated case reports [10, 11]. Moreover, is it uncertain how much routine screening would assist in mitigating these clinical outcomes. Significant AST and ALT abnormalities were very rare at baseline; and the predictive power of these tests for flares or withdrawal hepatitis is unknown. The rationale for screening for hepatitis virus is unclear to us.

Mild anaemia was very common, but significant anaemia (<8 g/dl), the threshold suggested in local guidelines as a contraindication to AZT, was very unusual, in keeping with other studies [24, 25]. In addition, neutropaenia was uncommon. This suggests that AZT may be used relatively safely in this group, without screening, although the role of the drug in resource-limited settings outside of second regimens is now largely confined to those with renal disease [2, 6].

Limitations of the study include the difficulties in generalising the findings to South Africa as a whole, given the urban nature and high percentage of Zimbabweans in the screened population. The findings, however, might apply to similar inner-city areas of the country, or even to parts of Zimbabwe. Also, participants in this study were on average relatively young and the creatinine clearance abnormalities may be more frequent among older patients. Excluding patients with peripheral neuropathy or exposure to antiretroviral drugs for preventing mother-to-child transmission of HIV may also limit generalisability of results, although unlikely to any meaningful degree. The use of community workers to identify potential participants, rather than using systematic sampling, may have incurred selection bias. Community workers could, for example, have been more likely to approach patients they knew, those of the same gender as themselves, or those they believed would be more likely to participate. Having been instructed to refer patients with a CD4 below 400 may have resulted in them purposely recruiting sicker looking patients. Finally, hepatitis B was screened using antigen testing, which may miss occult HBV infections [23, 26] and cases of delta virus, though the latter is very rare in South Africa [22, 27].

## Conclusion

The study suggests that there might be minimal value in routine laboratory screening to identify relative and absolute contraindications to the use of commonly used antiretrovirals in patients in inner-city Johannesburg. The value of the renal, hepatic and haematological screening tests prior to ART initiation should be carefully evaluated in further longitudinal studies and in a formal cost-effectiveness appraisal. Careful assessment of the value of screening tests, which can be expensive and delay initiation of ART, is needed when initiation guidelines are constructed, as once the tests have been recommended and they become standard of care, it is becomes difficult to remove those recommendations. In particular, the use of creatinine clearance testing in our population might be re-evaluated once the prospective data from this trial are available.

## Abbreviations

μL: Microlitre
3TC: Lamivudine
ALT: Alanine Aminotransferase
ART: Antiretroviral therapy
AST: Aspartate Aminotransferase
AZT: Zidovudine
CD4: Cluster of differentiation 4
EFV: Efavirenz
eGFR: Estimated glomerular filtration rate
FTC: Emtricitabine
Hb: Haemoglobin
HIV: Human immunodeficiency virus
IQ: Interquartile range
IU: International units
L: Litre
mL: Millilitre
n: Number
OI: Opportunistic infection
PMTCT: Prevention
SD: Standard deviation
TDF: Tenofovir
WHO: World Health Organisation
